# A US National Study of the Association Between Income and Ambulance Response Time in Cardiac Arrest

**DOI:** 10.1001/jamanetworkopen.2018.5202

**Published:** 2018-11-30

**Authors:** Renee Y. Hsia, Delphine Huang, N. Clay Mann, Christopher Colwell, Mary P. Mercer, Mengtao Dai, Matthew J. Niedzwiecki

**Affiliations:** 1 Department of Emergency Medicine, University of California, San Francisco; 2 Philip R. Lee Institute for Health Policy Studies, University of California, San Francisco; 3 Department of Pediatrics, University of Utah School of Medicine, Salt Lake City; 4 Mathematica Policy Research, Oakland, California

## Abstract

**Importance:**

Emergency medical services (EMS) provide critical prehospital care, and disparities in response times to time-sensitive conditions, such as cardiac arrest, may contribute to disparities in patient outcomes.

**Objectives:**

To investigate whether ambulance 9-1-1 times were longer in low-income vs high-income areas and to compare response times with national benchmarks of 4, 8, or 15 minutes across income quartiles.

**Design, Setting, and Participants:**

A retrospective cross-sectional study was performed of the 2014 National Emergency Medical Services Information System data in June 2017 using negative binomial and logistic regressions to examine the association between zip code–level income and EMS response times. The study used ambulance 9-1-1 response data for out-of-hospital cardiac arrest from 46 of 50 state repositories (92.0%) in the United States. The sample included 63 600 cardiac arrest encounters of patients who did not die on scene and were transported to the hospital.

**Main Outcomes and Measures:**

Four time measures were examined, including response time, on-scene time, transport time, and total EMS time. The study compared response times with EMS response time benchmarks for responding to cardiac arrest calls within 4, 8, and 15 minutes.

**Results:**

The study sample included 63 600 cardiac arrest encounters of patients (mean [SD] age, 60.6 [19.0] years; 57.9% male), with 37 550 patients (59.0%) from high-income areas and 8192 patients (12.9%) from low-income areas. High-income areas had greater proportions of white patients (70.1% vs 62.2%), male patients (58.8% vs 54.1%), privately insured patients (29.4% vs 15.9%), and uninsured patients (15.3% vs 7.9%), while low-income areas had a greater proportion of Medicaid-insured patients (38.3% vs 15.8%). The mean (SD) total EMS time was 37.5 (13.6) minutes in the highest zip code income quartile and 43.0 (18.8) minutes in the lowest. After controlling for urban zip code, weekday, and time of day in regression analyses, total EMS time remained 10% longer (95% CI, 9%-11%; *P* < .001), translating to 3.8 minutes longer in the poorest zip codes. The EMS response time to patients in high-income zip codes was more likely to meet 8-minute and 15-minute cutoffs compared with low-income zip codes.

**Conclusions and Relevance:**

Patients with cardiac arrest from the poorest neighborhoods had longer EMS times compared with those from the wealthiest, and response times were less likely to meet national benchmarks in low-income areas, which may lead to increased disparities in prehospital delivery of care over time.

## Key Points

**Question:**

Are ambulance 9-1-1 times longer in high-income compared with low-income areas, and how do response times compare with national benchmarks across income quartiles?

**Findings:**

In this US national, cross-sectional study of 63 600 patients with out-of-hospital cardiac arrest, EMS times remained 10% longer in the poorest areas and were more likely to meet 8-minute and 15-minute cutoffs in high-income areas.

**Meaning:**

Patients with cardiac arrest from the poorest neighborhoods had longer EMS times that were less likely to meet national benchmarks compared with those from the wealthiest neighborhoods, which may lead to increased disparities in the delivery of prehospital care over time.

## Teaser

This cross-sectional study investigates whether ambulance response time to 9-1-1 calls were longer in low-income vs high-income areas and compares response times with national benchmarks of 4, 8, or 15 minutes across income quartiles.

## Introduction

The Institute of Medicine has documented that unequal access to health care facilities and health care professionals serves as a principal contributor to health care disparities in vulnerable communities.^1^ In the wake of an increasing number of hospital and emergency department shutdowns, poorer neighborhoods and vulnerable populations have had even less access to care.^2,3,4^ Prehospital care has been shown to be an essential component of health care access,^5,6,7^ and emergency medical services (EMS) have a vital role in providing timely care to stabilize and transport sick patients.^6,7,8^ Anecdotally, the recent and abrupt closures and bankruptcy of privately owned ambulance companies have left patients stranded.^9,10^ The repercussions of the loss of these services may be particularly detrimental in low-income communities, which rely more heavily on prehospital care and have a higher increased incidence of severe, life-threatening illnesses.^11,12,13,14,15^

Few studies document the association between socioeconomic status (SES) and availability of prehospital care. While the evaluation of EMS care requires the consideration of many different quality measures, access and timeliness of care, as measured by ambulance response times, have long been reported across EMS systems and act as meaningful benchmarks to the public. Few studies have examined disparities in prehospital care; for example, one reported longer response and transport times for suspected patients with stroke from low-income areas, defined by the percentage of residents living below the household income poverty threshold in the United States.^16^ Another study showed shorter intervals for out-of-hospital transport in patients with chest pain from higher-income neighborhoods in Canada,^17^ and a third study focused solely on a county in California.^18^ However, the association between ambulance response and transport times and zip code–level income for time-sensitive conditions across the United States has not been widely studied.

Using time benchmarks adopted by many EMS systems, specifically response time, on-scene time, and transport time, this study sought to fill a critical gap in knowledge by examining whether socioeconomic disparities are associated with longer ambulance response and transport times for out-of-hospital cardiac arrest.^19,20^ Previous literature on the management of out-of-hospital cardiac arrest, the importance of early cardiopulmonary resuscitation (CPR) and defibrillation for improved outcomes,^21^ and the documentation of decreased survival by 7% to 10% per minute without critical intervention had defining roles in the development of many current national and regional benchmarks.^15,19,22,23^ While several other prehospital metrics exist along the chain of survival in out-of-hospital cardiac arrest, this particular disease state may serve as a proxy for other critical conditions that rely on timely prehospital recognition and treatment, such as respiratory distress, stroke, and trauma. To our knowledge, this is the first nationwide study to use these benchmarks in evaluating disparities in EMS times between patients with cardiac arrest from high-income and low-income US neighborhoods.

## Methods

### Setting and Study Population

We used the 2014 data (the most recent as of June 2017, the time of the analysis) from the National Emergency Medical Services Information System (NEMSIS), a voluntary national registry of EMS activations funded by the National Highway Traffic Safety Administration. We performed a retrospective cross-sectional study to estimate the association between the median income by zip code and EMS response times for responding to 9-1-1 calls and transporting patients with cardiac arrest to the hospital. Zip codes are 5-digit codes used by the US mailing system and refer to specific zones that may not necessarily conform to the limits of a city (ie, some cities, especially large ones, can have multiple zip codes, while others can share or have overlapping zip codes). NEMSIS provides a compilation of standardized EMS patient care reports (PCRs), which include patient demographics, clinical information, interventions performed, dispatch times, and transport data. The PCRs are obtained from local EMS agencies throughout the reporting state and submitted by state repositories. The University of California, San Francisco institutional review board deemed this study exempt from review. This study did not require informed consent from study participants because our data do not contain identifiable information and the study participants cannot be contacted. This study followed the Strengthening the Reporting of Observational Studies in Epidemiology (STROBE) reporting guideline.

NEMSIS data include 2497 of the 3144 counties found in the United States, and there are no election criteria associated with NEMSIS participation. The NEMSIS project seeks a complete census of all ground response EMS activations occurring in the United States. Air medical transports, critical care transports, interfacility transports, and community paramedicine visits are less likely to be included in the NEMSIS data set. Nevertheless, most states require (or expect) all EMS-related activations to be included in NEMSIS. Forty-six of 50 state repositories (92.0%) in the United States contributed data in 2014 to NEMSIS (Massachusetts, Texas, Louisiana, and Delaware did not contribute), with a mean of 79% (range, 18%-100%) of EMS agencies within each contributing state reporting EMS activation data (eFigure in the Supplement).^24^ Agencies submitting data based on the NEMSIS standard, which requires that records be collected electronically and submitted using national standards, are likely to submit 100% of records using the same approach. The EMS software commonly uses data validation rules when EMS providers are completing a PCR. Once records are submitted to the national registry, audit filters assess more than 400 validation rules and send a data quality report back to the EMS state offices.

We included 9-1-1 EMS activations for cardiac arrest, as indicated by 9-1-1 dispatch reports for patients who did not die on scene and were transported to the hospital. We chose to focus on cardiac arrests in particular because the life-threatening condition has a high mortality rate; requires immediate attention; is among the most easily and consistently identifiable, time-critical prehospital conditions; and has largely influenced benchmarks developed for EMS response times.^19,21,25,26^ Certainly, there are other time-sensitive conditions, such as blunt or penetrating trauma, for example, that would similarly require immediate attention but have been shown in prior work to occur less frequently within the patient’s zip code.^27^ Cardiac arrest has been shown to have a low rate of geographic discordance between patient residential zip code and incident location,^27^ and therefore, cardiac arrest served as a more appropriate time-sensitive condition for the purposes of this study. In addition, we chose to exclude conditions other than cardiac arrest for this analysis because we recognized that there might be other confounders associated with ambulance demand in communities with low vs high SES, and time as a proxy for quality of care was not as clinically relevant in lower-acuity conditions.

The NEMSIS data set consists of EMS activations and does not contain a registry of patients receiving care. Therefore, multiple emergency resources may have responded to the same 9-1-1 call, potentially resulting in multiple PCR submissions to respective state data repositories and subsequently the NEMSIS repository. To minimize inclusion of multiple activations for the same incident, we grouped all EMS activations using a single incident identification for EMS responses to a 9-1-1 call for cardiac arrest on the same date to the same zip code within 10 minutes of one another. We used the minimum response time among submitted PCRs as the time it took to reach the patient. We excluded EMS responses to mass casualty incidents, air or water rescues, and EMS transports that did not deliver the patient to a hospital.

### Outcome Measures

The primary outcome of our analysis included intervals between key points in the EMS encounter. We examined the following 4 time measures: (1) time between EMS dispatch to EMS arrival at the patient’s location (response time), (2) time between EMS arrival to EMS departure from the scene (on-scene time), (3) time between EMS departure to EMS arrival at the destination hospital (transport time), and (4) total EMS time. While a large emphasis of EMS reporting has traditionally been placed on reporting response times, we believe that reporting total EMS time as a sum of EMS response, care, and transport times more accurately accounts for any environmental confounding factors, such as scene safety, building access, and distance from a definitive care site. To remove potential data entry errors, we dropped any intervals lasting longer than 24 hours and dropped the top 0.1% with the longest intervals for response time, on-scene time, and transport time of the remaining observations. We compared the mean length of time in minutes using the lowest and highest income quartiles.

Many EMS systems have adopted benchmarks to arrive on scene in less than 4, 8, and 15 minutes, with aims to deliver early CPR and defibrillation by trained professionals based on evidence by Eisenberg et al^21^ and others,^15,20^ which demonstrated a survival benefit of CPR performed within 5 minutes and defibrillation within 9 minutes of collapse for patients with cardiac arrest. In this study, we compared response times with the same EMS benchmarks for responding to cardiac arrest calls within 4, 8, and 15 minutes. We used the industry convention in which arrival on scene within 8 minutes, for example, is defined as arrival in 8 minutes 59 seconds or less.

### Variables

Our primary independent variable was the income quartile for the zip code of the reported incident. We ranked zip codes by US Census–reported median household income in the past 12 months (in 2009 inflation-adjusted dollars) (eTable 1 in the Supplement) and divided them into quartiles. We created indicators for each quartile except for the highest income quartile (reference group).

To obtain information on cost of living, we calculated the mean fair market rent by county for a standard 2-bedroom residence using county-level fair market rent information from the US Department of Housing and Urban Development. For our population density variable, we linked EMS data to the US Census population density measure and created population density quartiles by zip code.

### Statistical Analysis

We performed all statistical analyses using a software program (Stata, version 14; StataCorp LP). Because of the nature of discrete, nonzero, and skewed distribution of the duration data, as well as the study design of measuring time to completion for each interval, we used negative binomial regression models to analyze the association between zip code income quartiles and EMS intervals. All outcomes are reported as incident rate ratios. To measure the association between zip code income and arrival by benchmark times of less than 4, 8, or 15 minutes, we used logistic regression models and reported the marginal effects. Outcomes are reported as minutes. The regressor of interest in all regressions was an indicator for low median income within the zip code. All models controlled for urban area, day of week, time of day, and US Census region. All hypothesis tests were 2-sided. We calculated 95% CIs for all regression estimates and indicated levels of significance at *P* < .10, *P* < .05, and *P* < .01 in the tables.

We performed 3 sets of sensitivity analyses. In our first set of sensitivity checks, we restricted our sample to zip codes in urban areas to exclude possible confounding factors in rural areas. We made an intentional decision a priori to exclude patient-level demographics because we did not want to “justify” disparities in response time due to these factors and certain area-level characteristics. However, in our second and third sets of sensitivity analyses, we included additional models to control for these variables as a demonstration of what can happen with the inclusion of collinear variables and what we believe to be inappropriate variables for answering this particular question (eg, population density, which controls out the demand for services). In our second set, we included additional controls for network driving distance between the incident zip code centroid and the destination hospital, patient-level demographics of age, race/ethnicity, sex, and health insurance coverage (for the 29.4% of the sample that included patient demographics), and interactions between urbanicity and time of day. In our third set, we used random-effects models and included patient-level demographics and area-level characteristics (population density and cost of living).

## Results

### Characteristics of High-Income and Low-Income Areas

We analyzed 63 600 cardiac arrest encounters of patients (mean [SD] age, 60.6 [19.0] years; 57.9% male) in the United States in which EMS responded and transported patients with cardiac arrest to the hospital. As summarized in Table 1, high-income areas had greater proportions of white patients (70.1% vs 62.2%; *P* < .001), male patients (58.8% vs 54.1%; *P* < .001), privately insured patients (29.4% vs 15.9%; *P* < .001), and uninsured patients (15.3% vs 7.9%; *P* < .001), while low-income areas had a greater proportion of Medicaid-insured patients (38.3% vs 15.8%; *P* < .001). A greater proportion of EMS activations in high-income areas occurred in urban zip codes compared with low-income areas (93.3% vs 61.4%; *P* < .001), with those in low-income areas requiring a slightly longer mean driving distance (6.77 vs 6.08 miles; *P* < .001). Our findings showed no significant differences between high-income and low-income areas in terms of weekday and weekend calls; however, low-income areas had a slightly higher proportion of calls earlier in the day during 7 am to 3 pm (40.4% vs 36.3%; *P* < .001).

**Table 1.** Summary Statistics of High-Income vs Low-Income Communities^**a**^

Variable	Mean (SD)		Difference	*P* Value
	High Income (n = 37 550)	Low Income (n = 8192)		
**Patient Characteristics**^**b**^				
Age, y	60.82 (19.55)	59.84 (17.29)	0.98	<.001
White race/ethnicity	0.70 (0.46)	0.62 (0.48)	0.08	<.001
Male	0.59 (0.49)	0.54 (0.50)	0.05	<.001
Health insurance				
Private	0.29 (0.46)	0.16 (0.37)	0.14	<.001
Medicaid	0.16 (0.37)	0.38 (0.49)	−0.22	<.001
Medicare	0.37 (0.48)	0.35 (0.48)	0.02	.02
Uninsured	0.15 (0.36)	0.08 (0.27)	0.07	<.001
Other	0.02 (0.15)	0.03 (0.16)	0.00	.18
**Response Characteristics**^**c**^^,^^**d**^				
Urban zip code	0.93 (0.25)	0.61 (0.49)	0.32	<.001
Driving distance, incident to hospital miles	6.08 (7.30)	6.77 (13.65)	−0.69	<.001
Weekday	0.72 (0.45)	0.72 (0.45)	0.00	.87
Weekend	0.72 (0.45)	0.72 (0.45)	0.00	.87
7 am to 3 pm	0.36 (0.48)	0.40 (0.49)	−0.04	<.001
3 pm to 11 pm	0.37 (0.48)	0.33 (0.47)	0.04	<.001
11 pm to 7 am	0.26 (0.44)	0.27 (0.44)	0.00	.73
Northeast	0.37 (0.48)	0.47 (0.50)	−0.10	<.001
Midwest	0.18 (0.38)	0.06 (0.24)	0.12	<.001
South	0.33 (0.47)	0.46 (0.50)	−0.13	<.001
West	0.12 (0.33)	0.02 (0.12)	0.10	<.001
Time measure, min				
Response time	8.24 (4.46)	9.08 (5.67)	−0.84	<.001
On-scene time	18.70 (9.34)	21.91 (13.35)	−3.20	<.001
Transport time	10.54 (7.31)	12.04 (9.35)	−1.50	<.001
Total EMS time	37.49 (13.61)	43.02 (18.83)	−5.54	<.001
Response time benchmarks, min				
<4	0.31 (0.46)	0.30 (0.46)	0.01	<.001
<8	0.78 (0.41)	0.72 (0.45)	0.06	<.001
<15	0.97 (0.18)	0.93 (0.26)	0.04	<.001

Abbreviation: EMS, emergency medical services.

^a^ The highest median zip code income quartile ranges from $57 502 to $113 313, and the lowest median zip code income quartile ranges from $20 250 to $42 642. Percentages may not sum to 100% because of rounding. The column showing the difference between high-income and low-income areas may not always exactly reflect the difference because of rounding.

^b^ Only 12 288 of 37 550 patients (32.7%) from high-income zip codes and 4647 of 8192 patients (56.7%) from low-income zip codes had all demographic data collected.

^c^ Weekday and weekend are reported in the summary table, but individual day indicators are included in the regression. Hours are reported in intervals in the summary table, but hour indicators are included in the regression (eg, 12 am to 1 am).

^d^ US Census divisions are reported as regions in the summary table, but individual divisions are included in the regression. See eTable 1 in the Supplement for US Census division and region definitions.

### Ambulance Transport Times for High-Income and Low-Income Areas

**Figure 1.**
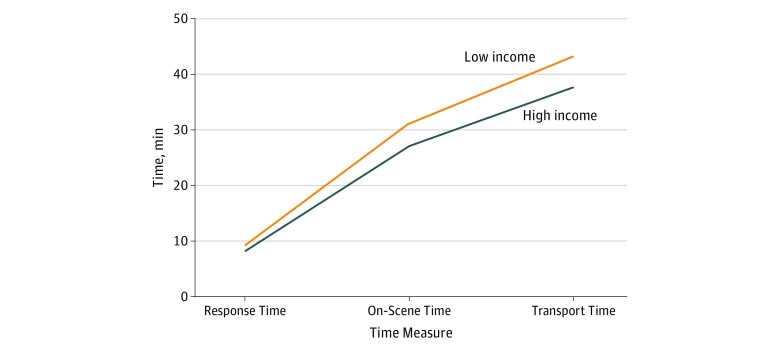
Income and Cumulative Emergency Medical Services Time The highest median zip code income quartile ranges from $57 502 to $113 313, and the lowest median zip code income quartile ranges from $20 250 to $42 642.

The mean (SD) total EMS time was 37.5 (13.6) minutes in the highest zip code income quartile and 43.0 (18.8) minutes in the lowest zip code income quartile (difference, −5.5 minutes; *P* < .001) (Table 1), with slightly longer subcategories (response time, on-scene time, and transport time) for low-income areas, as shown in Figure 1. We summarize in Table 2 that, after controlling for urban zip code, weekday, and time of day, total EMS time for low-income zip codes was 10% (or 3.8 minutes) longer (95% CI, 9%-11%; *P* < .001) compared with that of the wealthiest neighborhoods. Furthermore, we found that response time was 4% (or 0.3 minutes) longer (95% CI, 2%-6%; *P* < .001), on-scene time was 15% (or 2.8 minutes) longer (95% CI, 13%-17%; *P* < .001), and transport time was 6% (or 0.6 minutes) longer (95% CI, 4%-8%; *P* < .001) for low-income compared with high-income zip codes.

**Table 2.** Negative Binomial Regression Analysis of Zip Code Income Quartiles and Intervals During EMS Response Among 63 600 Cardiac Arrest Encounters^a^

Zip Code Income Quartile	Response Time^b^		On-Scene Time^c^		Transport Time		Total EMS Time^d^	
	IRR (95% CI)	*P* Value	IRR (95% CI)	*P* Value	IRR (95% CI)	*P* Value	IRR (95% CI)	*P* Value
1, Lowest	1.04 (1.02-1.06)^e^	<.001	1.15 (1.13-1.17)^e^	<.001	1.06 (1.04-1.08)^e^	<.001	1.10 (1.09-1.11)^e^	<.001
2	1.00 (0.98-1.02)	.01	0.96 (0.94-0.98)^e^	.01	0.97 (0.95-0.99)^e^	.01	0.97 (0.96-0.98)^e^	.01
3	1.02 (1.01-1.03)^e^	.004	1.00 (0.99-1.01)	.88	1.06 (1.04-1.07)^e^	<.001	1.02 (1.01-1.03)^e^	<.001
4, Highest	1 [Reference]	NA	1 [Reference]	NA	1 [Reference]	NA	1 [Reference]	NA

Abbreviations: EMS, emergency medical services; IRR, incident rate ratio; NA, not applicable.

^a^ Incident rate ratios from negative binomial regression models are reported. Income quartile 1 ranges from $20 250 to $42 642. Income quartile 2 ranges from $42 642 to $49 135. Income quartile 3 ranges from $49 135 to $57 502. Income quartile 4 ranges from $57 502 to $113 313. Controls were included for urban zip code, weekday, time of day, and US Census region. No patient-level controls were included. See eTable 2 in the Supplement for results using urban zip code only.

^b^ In calls with more than 1 EMS response unit, response time is calculated from the minimum of all responders.

^c^ On-scene time includes the time from when the first responder arrived at the patient to when the patient was transported from the scene.

^d^ Total EMS time includes time from dispatch to hospital (the sum of the first 3 columns).

^e^ Statistical significance denoted by *P* < .01.

**Figure 2.**
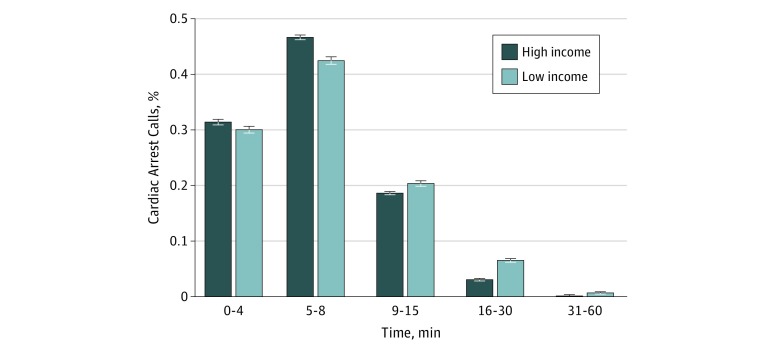
Distribution of Emergency Medical Services Response Times for 63 600 Cardiac Arrest Encounters The highest median zip code income quartile ranges from $57 502 to $113 313, and the lowest median zip code income quartile ranges from $20 250 to $42 642. Error bars indicate 95% CIs.

Most cardiac arrest EMS activations did not reach the incident scene within 4 minutes, with only 31.4% in high-income zip codes and 30.0% in low-income zip codes (*P* < .001) meeting the benchmark (Table 1). However, a higher proportion of EMS responses to patients in high-income zip codes met the 8-minute and 15-minute marks (78.1% and 96.7%, respectively) compared with low-income zip codes (72.4% and 92.7%, respectively) (*P* < .001 for both comparisons). Figure 2 shows a complete distribution of EMS response times. We estimate in Table 3 the likelihood of meeting the benchmarks, and found that EMS responses to low-income zip codes were less likely to meet the 15-minute benchmark (−1 percentage point; 95% CI, −2 to −1 percentage points; *P* < .001).

**Table 3.** Logistic Regression Analysis of Zip Code Income Quartiles and Response Time Among 63 600 Cardiac Arrest Encounters^a^

Zip Code Income Quartile	Response Time, min^b^					
	<4		<8		<15	
	Marginal Effects (95% CI)	*P* Value	Marginal Effects (95% CI)	*P* Value	Marginal Effects (95% CI)	*P* Value
1, Lowest	0.00 (−0.01 to 0.02)	.73	−0.01 (−0.02 to 0.00)^c^	.08	−0.01 (−0.02 to −0.01)^d^	<.001
2	0.00 (−0.01 to 0.02)	.46	−0.04 (−0.05 to −0.02)^d^	<.001	−0.01 (−0.02 to −0.01)^d^	<.001
3	−0.01 (−0.02 to −0.00)^e^	.04	−0.02 (−0.03 to −0.01)^d^	<.001	−0.01 (−0.01 to −0.00)^d^	.004
4, Highest	0 [Reference]	NA	0 [Reference]	NA	0 [Reference]	NA

Abbreviation: NA, not applicable.

^a^ Marginal effects from logistic regression models are reported. Income quartile 1 ranges from $20 250 to $42 642. Income quartile 2 ranges from $42 642 to $49 135. Income quartile 3 ranges from $49 135 to $57 502. Income quartile 4 ranges from $57 502 to $113 313. Controls were included for urban zip code, weekday, time of day, and US Census region. No patient-level controls were included. See eTable 3 in the Supplement for results using urban zip code only.

^b^ In calls with more than 1 emergency medical services response unit, response time is calculated from the minimum of all responders.

^c^ Statistical significance denoted by *P* < .10.

^d^ Statistical significance denoted by *P* < .01.

^e^ Statistical significance denoted by *P* < .05.

In sensitivity analyses, we restricted the sample to patients in urban zip codes, and our results remained robust (eTable 2 and eTable 3 in the Supplement). The disparity in the mean total response time attenuated slightly from 10% to 9% longer in low-income zip codes. When we included network driving distance between the incident zip code centroid and the destination hospital, the results remained significant for the estimated mean response time and total EMS time benchmarks (eTable 4 and eTable 5 in the Supplement). In our “overcontrolled” model that includes patient-level and area-level factors, we still found that total EMS time remained longer for the lowest-income zip codes compared with the highest (eTable 6 in the Supplement). However, the lowest- and highest-income zip codes did not differ significantly in likelihoods of meeting 4-minute, 8-minute, and 15-minute benchmarks (eTable 7 in the Supplement).

## Discussion

Our study demonstrates that total EMS time for cardiac arrest incidents is longer and that a lower proportion of 9-1-1 calls meets national ambulance response time benchmarks in low-income compared with high-income neighborhoods. We found a persistent and significant time difference between high-income and low-income zip codes, even after controlling for common EMS system demand indicators, such as weekday and time of day to account for traffic. To our knowledge, our study is one of the first to evaluate the association of zip code income level with EMS response and transport times. Our findings are particularly concerning given the time sensitivity of conditions like cardiac arrest in which the heart has ceased functioning and immediate medical care is required to restore function and circulation. In fact, a recent study^28^ showed that even a 4.4-minute delay is associated with a 13% increase in 30-day mortality. This finding is similar to another landmark study^8^ performed using EMS response times and mortality, which documented a 17% increase in 1-year mortality from a 1-minute delay. Furthermore, given that rates of out-of-hospital cardiac arrest are higher,^29,30^ bystander CPR is less likely,^1,13,31,32,33,34,35,36^ and post–cardiac arrest survival is lower^30,37^ in these neighborhoods, our findings of increased transport times for low-income communities are particularly alarming. While some may argue that improvement in cardiac arrest survival will come from interventions made earlier along the chain of survival, including bystander CPR and defibrillation, differences in EMS times for cardiac arrest EMS activations may serve as a proxy to understand possible disparities in prehospital care for poor neighborhoods.

While our main model intentionally excluded socioeconomic factors, such as age, race/ethnicity, sex, and health insurance coverage, we performed additional analyses (eTables 6 and 7 in the Supplement) to demonstrate how inclusion of these factors can attenuate positive findings. There is a much larger debate on the national scene about controlling for these potential “explanatory” factors, as evidenced by a rigorous and public discussion within the National Quality Forum about when performance measures should be adjusted for sociodemographic factors and when they should not.^38^ This debate has been described as “politically controversial and…a thorny methodological issue.”^39^^(p348)^ The panel concluded that “blanket adjustment of performance measures also would not be appropriate,” with a recommendation for “measure-by-measure” determination to prevent creating lower standards of care based on socioeconomic disadvantage.^40^^(p2615)^ Therefore, by the National Quality Forum’s criteria, adjusting EMS time with socioeconomic factors would not be appropriate because certain factors, such as age, race/ethnicity, and health insurance coverage, are unknown before arrival for a call and should have little association with the timeliness of EMS services.

Our findings are consistent with 2 other studies^16,17^ looking at SES and EMS times, except that we found on-scene time to be the longest interval for both high-income and low-income groups. While on-scene delays have not been well studied, some factors may include reaching patients in high-rise buildings in urban areas and establishing scene safety.^41,42,43^ For example, prehospital health care professionals responding to patients living in low-income zip codes residing in dense housing complexes could also encounter more logistic difficulties in identifying the proper housing unit or could encounter language barriers, which are present less often in high-income neighborhoods. Although debates on duration of resuscitation and variations with infield termination practices continue, many EMS systems place emphasis on staying at the scene until the return of spontaneous circulation, which may explain longer on-scene times.^44,45,46,47^ Given that some types of cardiac arrest (eg, ventricular tachycardia or ventricular fibrillation) are more amenable to achieving the return of spontaneous circulation, low SES–related factors associated with non–ventricular tachycardia/ventricular fibrillation rhythms, such as drug use, could explain longer resuscitation duration and worse survival outcomes.^48^

Our results differ from those of a recent study^18^ of ambulance response times within a single county, which found that the median response times (from dispatch to the scene only) to poorer neighborhoods were shorter than response times to wealthier neighborhoods. That study included population density in its modeling, which, as noted earlier, would be expected to decrease any potential associations because more people in a given area indicate a greater demand for EMS services, as appropriately should be the case. In addition, as acknowledged in the study, its single county’s lower SES neighborhoods were more urban than higher SES areas, as well as closer to hospitals. Finally, the study did not examine on-scene or transport to hospital times. In our national study, distance traveled by EMS was slightly longer in low-income neighborhoods compared with high-income neighborhoods. This finding is likely due to an imbalance of initial hospital allocations as well as to an increasing number of emergency department and hospital closures in lower-income areas. These closures can lead to longer EMS times^3,4,49^ because existing emergency departments become more overcrowded, leading to frequently diverted ambulances and further increasing transportation times.^50^ When one arm of the health care system is incapacitated, there can be bilateral rippling consequences upstream on prehospital services and overall access to the health care system and downstream on patient outcomes.

In addition to overall availability of hospitals, current trends in the locations of hospitals specializing in cardiac conditions may partially explain longer distances and EMS times for low-income communities.^51^ Patients with cardiac arrest have been shown to have better outcomes if treated in specialized cardiac arrest centers, and EMS protocols frequently reflect these decisions, even if the center is farther away.^52^ Despite these recommendations, new centers with percutaneous coronary intervention (PCI) capabilities are concentrated among other existing centers and are located in wealthier and insured communities. In fact, while the number of PCI-capable hospitals grew 44% between 2001 and 2006, population access to PCI only increased by 1%, indicating a severe duplication of services.^53,54^ Recent work on timely access to PCI-capable facilities confirmed that low-income areas are disproportionately underserved.^55^

Simultaneously, the new, shifting landscape to privately owned ambulance companies may lead to a greater focus on profitability over public need, which could drive more companies to serve wealthier neighborhoods, potentially increasing total EMS time because poorer neighborhoods would have fewer ambulances and personnel to go around. In conjunction with decreasing EMS supply, emerging news of poorly staffed and underregulated ambulance companies declaring bankruptcy after privatization could further exacerbate total EMS time.^10^ Because low-income patients are more dependent on EMS for hospital transport, these reductions in prehospital services likely have disproportionate, detrimental downstream influences on the poor, exacerbating rather than alleviating health disparities.^56,57^

### Limitations

Our study has several limitations. First, NEMSIS is a registry of EMS activations rather than individual patients, leading to the existence of multiple PCRs associated with the same patient, which overtly centralized the median values and artificially reduced the width of reported 95% CIs. However, a subanalysis showed that multiple PCRs did not significantly alter our results. Second, EMS system performance metrics are not yet nationally standardized.^26^ While response time has been one of the main standards used to judge EMS performance, some systems stop the clock at arrival to the scene but not necessarily to the patient. Furthermore, the commonly cited benchmarks used in this article are primarily based on expert opinions because individual EMS system guidelines vary between organizations. Our findings could be considered conservative given that other studies call for more stringent benchmarks. For example, the Ontario Prehospital Advanced Life Support Study Group^19^ found that there was a steep increase in mortality past the first 5 minutes of response time without any interventions. Future studies could focus on addressing standard benchmarks to help guide infrastructure design and criteria for interval evaluation. Third, given that it is not possible to do this type of research as an experiment, it is always possible that there are other factors driving disparities. For example, some have suggested that ride-sharing services (eg, Uber) may reduce ambulance volume, and given that these services were rolled out to higher-value cities earlier than others,^58^ it is possible that there are other explanations beyond our variables that explain these differences. Fourth, cardiac arrests account for less than 1% of the 9-1-1 call volume, making it unclear whether these findings can be generalized to other types of high-acuity, time-sensitive EMS calls. At the same time, our findings are likely conservative given that cardiac arrest is considered one of the most time-critical conditions and necessitates emergent medical care.

## Conclusions

Our analysis demonstrated that EMS responding to low-income communities had a lower likelihood of meeting 8-minute and 15-minute national benchmarks compared with EMS responding to high-income communities and showed that the mean EMS response time, on-scene time, and transport time were longer in low-income communities, even after controlling for observable differences. Given that whether or not a patient survives cardiac arrest can depend on a matter of minutes, even small delays in EMS response times may negatively alter patient outcomes. Our findings are disturbing given that poorer neighborhoods have higher rates of disease and other structural disparities in health care access that further compound their risk for worse outcomes. Our study shows that these structural disparities begin as early as the initial EMS activation and the resulting services, which is an area previously more traditionally administered by public services and considered less vulnerable to market forces. Recent trends in the financing and delivery of prehospital care suggest that these disparities are likely to worsen unless fewer economically driven forces are introduced. Understanding where gaps exist can help guide improvements in policies and develop interventions to address prehospital care disparities and ultimately disparities in patient outcomes.

## Notes

Supplement. **eFigure.** States Participating in the National Emergency Medical Services Information System (NEMSIS) **eTable 1.** Census Regions **eTable 2.** Negative Binomial Regression Results Using Urban Zip Codes Only **eTable 3.** Logistic Regression Results, Marginal Effects Using Urban Zip Codes Only **eTable 4**. Negative Binomial Regression Results – Sensitivity Analysis **eTable 5.** Logistic Regression Results, Marginal Effects – Sensitivity Analysis **eTable 6.** Negative Binomial Regression Results With Random Effects **eTable 7.** Logistic Regression Results, Marginal Effects With Random Effects
