# Supplementary material for: A US National Study of the Association Between Income and Ambulance Response Time in Cardiac Arrest
Source: JAMA Netw Open. 2018 Nov 30;1(7):e185202. doi: 10.1001/jamanetworkopen.2018.5202 (PMC6324393; doi:10.1001/jamanetworkopen.2018.5202)
Supplement: Supplement. — eFigure. States Participating in the National Emergency Medical Services Information System (NEMSIS) eTable 1. Census Regions eTable 2. Negative Binomial Regression Results Using Urban Zip Codes Only eTable 3. Logistic Regression Results, Marginal Effects Using Urban Zip Codes Only eTable 4. Negative Binomial Regression Results – Sensitivity Analysis eTable 5. Logistic Regression Results, Marginal Effects – Sensitivity Analysis eTable 6. Negative Binomial Regression Results With Random Effects eTable 7. Logistic Regression Results, Marginal Effects With Random Effects [file jamanetwopen-1-e185202-s001.pdf]

Supplementary Online Content

Hsia RY, Huang D, Mann NC, et al. A US national study of the association between income and ambulance response time in cardiac arrest. *JAMA Netw Open*. 2018;1(7):e185202. doi:10.1001/jamanetworkopen.2018.5202

eFigure. States Participating in the National Emergency Medical Services Information System (NEMSIS)

eTable 1. Census Regions

eTable 2. Negative Binomial Regression Results Using Urban Zip Codes Only

eTable 3. Logistic Regression Results, Marginal Effects Using Urban Zip Codes Only

eTable 4. Negative Binomial Regression Results – Sensitivity Analysis

eTable 5. Logistic Regression Results, Marginal Effects – Sensitivity Analysis

eTable 6. Negative Binomial Regression Results With Random Effects

eTable 7. Logistic Regression Results, Marginal Effects With Random Effects

This supplementary material has been provided by the authors to give readers additional information about their work.

© 2018 Hsia RY et al. *JAMA Network Open*.

eFigure. States Participating in the National Emergency Medical Services Information System (NEMSIS)

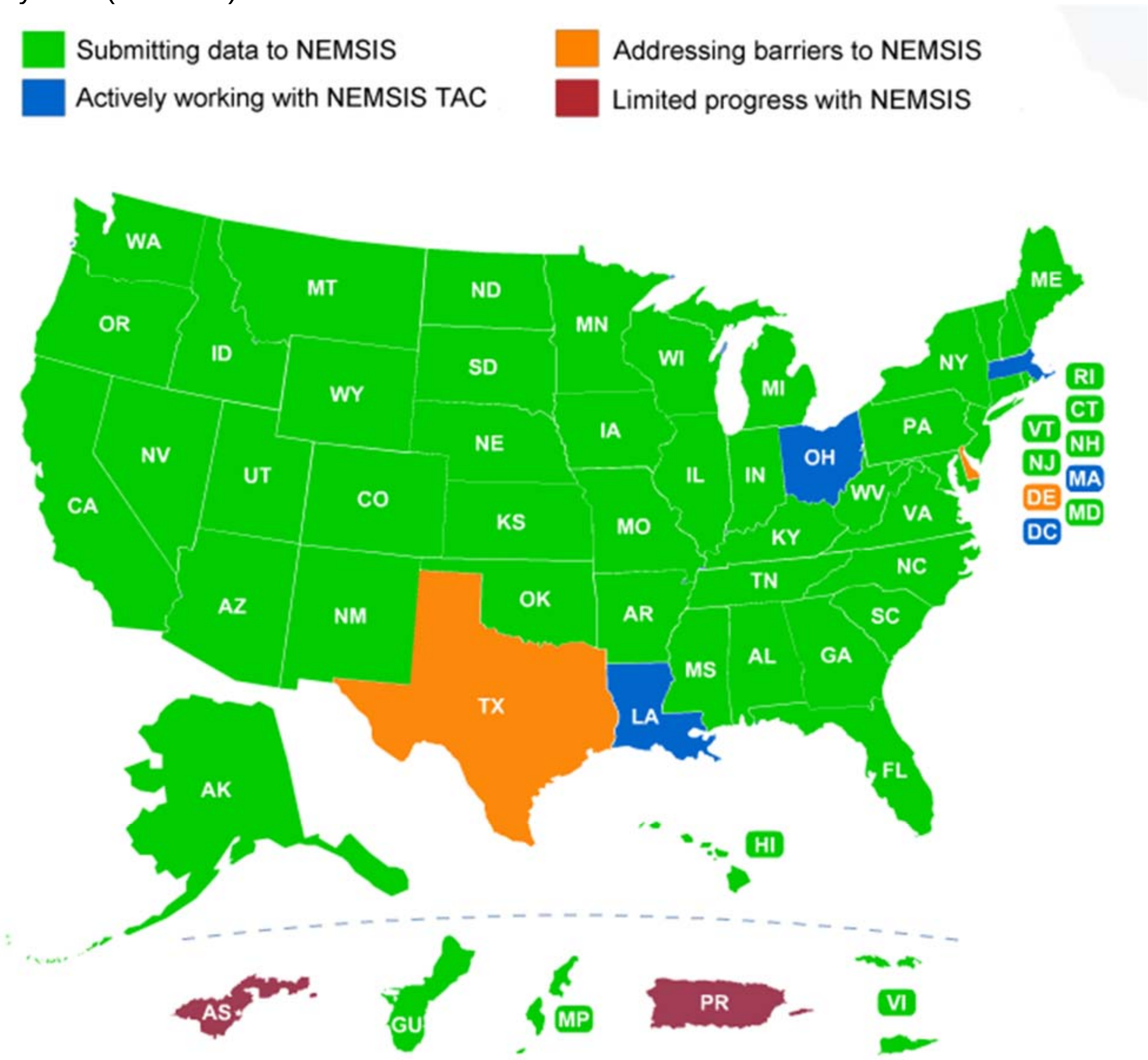

Abbreviations: TAC – Technical Assistance Center.

© 2018 Hsia RY et al. *JAMA Network Open*.

eTable 1. Census Regions

Region	Division	States
Northeast	New England	Connecticut, Maine, Massachusetts, New Hampshire, Rhode Island, Vermont
	Mid-Atlantic	Delaware, New Jersey, New York, Pennsylvania
Midwest	East North Central	Illinois, Indiana, Michigan, Ohio, and Wisconsin
	West North Central	Iowa, Kansas, Minnesota, Missouri, Nebraska, North Dakota, South Dakota
South	South Atlantic	Florida, Georgia, Maryland, North Carolina, South Carolina, Virginia, District of Columbia, West Virginia
	East South Central	Alabama, Kentucky, Mississippi, Tennessee
	West South Central	Arkansas, Louisiana, Oklahoma, Texas
West	Mountain	Arizona, Colorado, Idaho, Montana, Nevada, New Mexico, Utah, Wyoming
	Pacific	Alaska, California, Hawaii, Oregon, Washington

Source: https://www2.census.gov/geo/docs/maps-data/maps/reg_div.txt

© 2018 Hsia RY et al. *JAMA Network Open*.

eTable 2. Negative Binomial Regression Results Using Urban Zip Codes Only

Zip Code Income	Time to Patient	Time on Scene	Transport Time	Total Time
Quartile 1 (poorest)	1.01	1.14**	1.05**	1.09**
	[0.99, 1.03]	[1.12, 1.15]	[1.03, 1.08]	[1.07, 1.10]
Quartile 2	0.99	0.93**	0.94**	0.95**
	[0.97, 1.01]	[0.91, 0.96]	[0.92, 0.96]	[0.93, 0.96]
Quartile 3	1.01*	0.99+	1.07**	1.02**
	[1.00, 1.03]	[0.98, 1.00]	[1.06, 1.09]	[1.01, 1.03]
Quartile 4	1	1	1	1
	[Ref]	[Ref]	[Ref]	[Ref]
N	53,516	53,516	53,516	53,516

Notes: Incident rate ratios from negative binomial regression model reported. Income Quartile 1 ranges $20,250 - $42,642. Income Quartile 2 ranges $42,642 - $49,135. Income Quartile 3 ranges $49,135 - $57,502. Income Quartile 4 ranges $57,502 - $113,313. Controls included for time of day, day of week, and census region; no patient-level controls were included. In calls with more than one emergency medical services response unit, time to patient is calculated from the minimum of all responders. Time on scene includes the time from when the first responder arrived at the patient to when the patient was transported from the scene. Total time includes time from dispatch to hospital (the sum of the first three columns). Statistical significance denoted by + p<0.10, * p<0.05, ** p<0.01.

© 2018 Hsia RY et al. *JAMA Network Open*.

eTable 3. Logistic Regression Results, Marginal Effects Using Urban Zip Codes Only

	**Time to scene**		
Zip Code Income	<4 min	<8 min	<15 min
Quartile 1 (poorest)	0.01	0.03**	0.00
	[-0.00,0.03]	[0.01,0.04]	[-0.01,0.01]
Quartile 2	0.00	-0.04**	-0.01**
	[-0.01,0.02]	[-0.05,-0.03]	[-0.02,-0.00]
Quartile 3	-0.01**	-0.02**	-0.01**
	[-0.02,-0.00]	[-0.03,-0.01]	[-0.01,-0.00]
Quartile 4	0	0	0
	[Ref]	[Ref]	[Ref]
N	53,516	53,516	53,516

Notes: Results are reported as marginal effects from logistic regression. Income Quartile 1 ranges $20,250 - $42,642. Income Quartile 2 ranges $42,642 - $49,135. Income Quartile 3 ranges $49,135 - $57,502. Income Quartile 4 ranges $57,502 - $113,313. In calls with more than one emergency medical services response unit, time to scene is calculated from the minimum of all responders. Controls included for urban zip code, time of day, day of week, and census region; no patient-level controls were included. Statistical significance denoted by ** $p<0.01$.

© 2018 Hsia RY et al. *JAMA Network Open*.

eTable 4. Negative Binomial Regression Results – Sensitivity Analysis

	**Including network driving distance**				**Including network driving distance and patient demographics**				**Including network driving distance, patient demographics, and interaction of urban zip code and hour of day**			
Zip Code Income	Time to Patient	Time on Scene	Transport Time	Total Time	Time to Patient	Time on Scene	Transport Time	Total Time	Time to Patient	Time on Scene	Transport Time	Total Time
Quartile 1 (poorest)	1.03**	1.15**	1.06**	1.10**	1.04*	1.14**	1.00	1.08**	1.04*	1.14**	1.00	1.08**
	[1.02,1.05]	[1.13,1.16]	[1.04,1.08]	[1.09,1.11]	[1.01,1.07]	[1.10,1.17]	[0.97,1.04]	[1.06,1.10]	[1.01,1.07]	[1.10,1.17]	[0.97,1.04]	[1.06,1.11]
Quartile 2	1.00	0.95**	0.94**	0.96**	1.01	1.02	0.92**	0.99	1.01	1.03	0.92**	0.99
	[0.98,1.02]	[0.93,0.97]	[0.92,0.96]	[0.95,0.97]	[0.97,1.04]	[0.99,1.06]	[0.89,0.96]	[0.97,1.02]	[0.97,1.04]	[0.99,1.06]	[0.89,0.96]	[0.97,1.02]
Quartile 3	1.02**	1.00	1.04**	1.02**	1.01	1.02	1.03*	1.02**	1.01	1.02	1.03*	1.02**
	[1.00,1.03]	[0.99,1.01]	[1.02,1.05]	[1.01,1.02]	[0.99,1.03]	[0.99,1.04]	[1.00,1.05]	[1.01,1.03]	[0.99,1.03]	[0.99,1.04]	[1.00,1.05]	[1.01,1.04]
Quartile 4	1	1	1	1	1	1	1	1	1	1	1	1
	[Ref]	[Ref]	[Ref]	[Ref]	[Ref]	[Ref]	[Ref]	[Ref]	[Ref]	[Ref]	[Ref]	[Ref]
N	52,286	52,286	52,286	52,286	13,598	13,598	13,598	13,598	13,598	13,598	13,598	13,598

Notes: Incident rate ratios from the negative binomial regression model reported. Income Quartile 1 ranges $20,250 - $42,642. Income Quartile 2 ranges $42,642 - $49,135. Income Quartile 3 ranges $49,135 - $57,502. Income Quartile 4 ranges $57,502 - $113,313. In calls with more than one emergency medical services response unit, time to patient is calculated from the minimum of all responders. Time on scene includes the time from when the first responder arrived at the patient to when the patient was transported from the scene. Total time includes time from dispatch to hospital (the sum of the first three columns). Controls included for urban zip code, time of day, day of week, and census region. Additional controls included network driving distance between incident zip code centroid and destination hospital zip code centroid, patient demographics (age, sex, health insurance coverage, and white race), and interaction terms for urbanicity and hour of the day. Statistical significance denoted by * $p<0.05$, ** $p<0.01$.

© 2018 Hsia RY et al. *JAMA Network Open*.

eTable 5. Logistic Regression Results, Marginal Effects – Sensitivity Analysis

	**Including network driving distance**			**Including network driving distance and patient demographics**			**Including network driving distance, patient demographics, and interaction of urban zip code and hour of day**		
	Time to Scene			*Time to Scene*			*Time to Scene*		
Zip Code Income	<4 min	<8 min	<15 min	<4 min	<8 min	<15 min	<4 min	<8 min	<15 min
Quartile 1 (poorest)	0.00	-0.01*	-0.01**	0.01	-0.03*	-0.02**	0.01	-0.03*	-0.02**
	[-0.01, 0.01]	[-0.03, -0.00]	[-0.01, -0.00]	[-0.01, 0.04]	[-0.06, -0.01]	[-0.03, -0.01]	[-0.01, 0.04]	[-0.06, -0.01]	[-0.03, -0.01]
Quartile 2	0.00	-0.05**	-0.01**	0.04**	-0.04**	-0.01**	0.04**	-0.04**	-0.01**
	[-0.01, 0.01]	[-0.06, -0.03]	[-0.01, -0.01]	[0.01, 0.07]	[-0.07, -0.02]	[-0.02, -0.00]	[0.01, 0.07]	[-0.06, -0.02]	[-0.02, -0.00]
Quartile 3	-0.01*	-0.03**	-0.00*	0.00	-0.03**	-0.01*	0.00	-0.03**	-0.01*
	[-0.02, -0.00]	[-0.04, -0.02]	[-0.01, -0.00]	[-0.02, 0.02]	[-0.05, -0.01]	[-0.01, -0.00]	[-0.02, 0.02]	[-0.05, -0.01]	[-0.01, -0.00]
Quartile 4	0	0	0	0	0	0	0	0	0
	[Ref]	[Ref]	[Ref]	[Ref]	[Ref]	[Ref]	[Ref]	[Ref]	[Ref]
N	52,286	52,286	52,286	13,598	13,598	13,598	13,598	13,598	13,598

Notes: Results are reported as marginal effects from the logistic regression. Income Quartile 1 ranges $20,250 - $42,642. Income Quartile 2 ranges $42,642 - $49,135. Income Quartile 3 ranges $49,135 - $57,502. Income Quartile 4 ranges $57,502 - $113,313. In calls with more than one emergency medical services response unit, time to scene is calculated from the minimum of all responders. Controls included for urban zip code, time of day, day of week, and census region. Additional controls included network driving distance between incident zip code centroid and destination hospital zip code centroid, patient demographics (age, sex, health insurance coverage, and white race), and interaction terms for urbanicity and hour of the day. Statistical significance denoted by * p<0.05, ** p<0.01

© 2018 Hsia RY et al. *JAMA Network Open*.

eTable 6. Negative Binomial Regression Results With Random Effects

Zip Code Income	Time to Patient	Time on Scene	Transport Time	Total Time
Quartile 1 (poorest)	1.02	1.16**	1.02	1.09**
	[0.99, 1.05]	[1.12, 1.19]	[0.99, 1.06]	[1.07, 1.11]
Quartile 2	1.00	1.02	0.92**	0.99
	[0.96,1.04]	[0.99,1.06]	[0.89,0.96]	[0.97,1.01]
Quartile 3	1.01	1.00	1.02	1.01
	[0.98,1.03]	[0.98,1.03]	[1.00,1.05]	[1.00,1.03]
Quartile 4	1	1	1	1
	[Ref]	[Ref]	[Ref]	[Ref]
N	13598	13598	13598	13598

Notes: Incident rate ratios from the negative binomial regression model reported. Income Quartile 1 ranges $20,250 - $42,642. Income Quartile 2 ranges $42,642 - $49,135. Income Quartile 3 ranges $49,135 - $57,502. Income Quartile 4 ranges $57,502 - $113,313. Controls included for time of day, day of week, and census region; patient-level controls included age, insurance status, gender, race, and level of acuity. Area-level characteristics included population density and cost of living. In calls with more than one emergency medical services response unit, time to patient is calculated from the minimum of all responders. Time on scene includes the time from when the first responder arrived at the patient to when the patient was transported from the scene. Total time includes time from dispatch to hospital (the sum of the first three columns). Statistical significance denoted by ** p<0.01.

© 2018 Hsia RY et al. *JAMA Network Open*.

eTable 7. Logistic Regression Results, Marginal Effects With Random Effects

	**Time to scene**		
Zip Code Income	<4 min	<8 min	<15 min
Quartile 1 (poorest)	0.02+	0.00	0.00
	[-0.00,0.05]	[-0.02,0.03]	[-0.01,0.01]
Quartile 2	0.05**	-0.02	0.00
	[0.02,0.07]	[-0.04,0.01]	[-0.01,0.00]
Quartile 3	0.00	-0.01	0.00
	[-0.02,0.02]	[-0.03,0.01]	[-0.01,0.00]
Quartile 4	0	0	0
	[Ref]	[Ref]	[Ref]
N	13598	13598	13598

Notes: Results are reported as marginal effects from logistic regression. Income Quartile 1 ranges $20,250 - $42,642. Income Quartile 2 ranges $42,642 - $49,135. Income Quartile 3 ranges $49,135 - $57,502. Income Quartile 4 ranges $57,502 - $113,313. In calls with more than one emergency medical services response unit, time to scene is calculated from the minimum of all responders. Controls included for urban zip code, time of day, day of week, and census region; patient-level controls included age, insurance status, gender, race, and level of acuity. Area-level characteristics included population density and cost of living. Statistical significance denoted by + p<0.10, ** p<0.01.

© 2018 Hsia RY et al. *JAMA Network Open*.
